# Nociception in Chicken Embryos, Part I: Analysis of Cardiovascular Responses to a Mechanical Noxious Stimulus

**DOI:** 10.3390/ani13172710

**Published:** 2023-08-25

**Authors:** Larissa Weiss, Anna M. Saller, Julia Werner, Stephanie C. Süß, Judith Reiser, Sandra Kollmansperger, Malte Anders, Heidrun Potschka, Thomas Fenzl, Benjamin Schusser, Christine Baumgartner

**Affiliations:** 1 Center for Preclinical Research, TUM School of Medicine, Technical University of Munich, 81675 Munich, Germany; larissa.weiss@tum.de (L.W.); anna.saller@tum.de (A.M.S.); julia.werner@tum.de (J.W.); stephanie.suess@tum.de (S.C.S.); judith.reiser@tum.de (J.R.); 2 Clinic for Anesthesiology and Intensive Care, TUM School of Medicine, Technical University of Munich, 81675 Munich, Germany; s.kollmansperger@outlook.de (S.K.); malteanders@gmail.com (M.A.); thomas.fenzl@tum.de (T.F.); 3 Institute of Pharmacology, Toxicology, and Pharmacy, Ludwig-Maximilians-Universität München, 80539 Munich, Germany; potschka@pharmtox.vetmed.uni-muenchen.de; 4 Reproductive Biotechnology, TUM School of Life Sciences, Technical University of Munich, 85354 Freising, Germany; benjamin.schusser@tum.de; 5 Veterinary Faculty, Ludwig-Maximilians-Universität München, 80539 Munich, Germany

**Keywords:** blood pressure, heart rate, nociception, pain, chicken embryo, development, *Gallus gallus domesticus*, poultry

## Abstract

**Simple Summary:**

Chicken embryos are frequently not protected by animal welfare laws. However, they are used in various research areas, and male embryos are commonly killed in food production as an alternative to culling day-old chicks. Increasing knowledge regarding the onset of nociception and pain perception in chicken embryos is fundamental for animal welfare protection. The aim of this exploratory study was to further narrow down the period when chicken embryos acquire the capacity for nociception. Therefore, changes in blood pressure and heart rate after the introduction of a noxious stimulus were assessed during the embryonic development of chickens. Embryos from 16 days of incubation onward showed cardiovascular changes after a noxious mechanical stimulus was introduced at the base of the beak, indicating a nociceptive response.

**Abstract:**

Although it is assumed that chicken embryos acquire the capacity for nociception while developing in the egg, an exact time point has not yet been specified. The present research was an exploratory study aiming to determine when the capacity of nociception emerges during embryonic development in chickens. Changes in blood pressure and heart rate (HR) in response to a noxious mechanical stimulus at the base of the beak versus a light touch on the beak were examined in chicken embryos between embryonic days (EDs) 7 and 18. Mean arterial pressure (MAP) was the most sensitive parameter for assessing cardiovascular responses. Significant changes in MAP in response to a noxious stimulus were detected in embryos at ED16 to ED18, whereas significant changes in HR were observed at ED17 and ED18. Infiltration anesthesia with the local anesthetic lidocaine significantly reduced the response of MAP on ED18, so the measured cardiovascular changes may be interpreted as nociceptive responses.

## 1. Introduction

In present times, animal welfare has increasingly become the focus of public attention regarding farm and laboratory animals. Consequently, the culling of male day-old chickens for economic reasons is increasingly questioned. A large proportion of the male offspring in the layer industry are killed after hatching, as the fattening of male layer-type chickens is not economically profitable [1]. In the EU, 330 million male chicks are killed annually through maceration or gassing [2], which is currently the subject of a major discussion. Germany and France have already adapted their laws and banned the killing of male day-old chicks for economic reasons, although there is not yet an EU-wide regulation [3]. As an alternative, *in ovo* sex determination with subsequent killing of male embryos is already being practiced [4]. However, it is important for animal welfare reasons and for the public acceptance of *in ovo* sex determination that related culling be conducted at an early stage of development when nociception and the perception of pain are not yet possible [4,5]. According to current knowledge, methods of *in ovo* sex determination are reliably applicable from the 9th day of incubation at the earliest [1]. Methods that can be applied in the first trimester of embryonic development are still in development under laboratory conditions [4].

Furthermore, chicken embryos are of great importance for biomedical research because of the advantages they provide in terms of fast growth and because of their good accessibility in various research areas, such as developmental biology, toxicology, cancer research and drug development [6,7]. Under European regulations, interventions and treatments on chicken embryos are not considered animal experiments and even count as a replacement method in the context of the 3R principles [8]. At this time, there are no regulations regarding anesthesia and analgesia of chicken embryos during painful interventions [6,8]. Greater clarity regarding the period during which chicken embryos are capable of nociception and pain sensation would lead to improved animal welfare in research.

In pain research, a fundamental distinction is made between nociception and the perception of pain [9]. Although nociception is the detection of a potentially tissue-damaging stimulus and its transmission by the nociceptive component of the nervous system [10,11], pain is characterized by a subjective, conscious sensory perception, usually triggered by nociception [12,13]. Nociception and pain are progressive adaptive processes that gradually develop throughout the fetal period [14]. It is considered confirmed that the chicken embryo acquires the capacity for nociception at some point during the 21-day developmental period in the egg [8,15]. However, the question of the exact time point at which nociception or even pain sensation can be presumed is controversial. In several publications, researchers agree that nociception and pain perception are not possible in the first trimester of embryonic development in the chicken [4,15]. A requirement for the ability to perceive pain is the existence of functional pathways that enable the transmission of stimuli to the brain [12,14]. Although the first sensory afferent nerve fibers develop on incubation day 4, the closure of multisynaptic reflex arcs does not occur until day 7 [16,17,18]. It is described in the literature that the chicken embryo develops a functional brain on day 13 [15,19]. However, it is only confirmed that the brain does not show any electrical activity until 6.5 days of incubation [20]. Pain sensation is therefore considered impossible up to incubation day 7, but beyond that, no specific time point can be defined from which the chicken embryo is capable of nociception and pain sensation [4,15].

Because self-reporting, which is the gold standard in humans to detect pain [21], is not possible as a direct method of pain evaluation in animals, indirect methods such as the alteration of physiological and behavioral parameters must be resorted to [22]. Changes in heart rate (HR) and blood pressure are therefore used as clinical indicators of nociception and pain [23].

This study is part of a comprehensive study in which the nociceptive ability of chicken embryos was investigated using cardiovascular parameters, behavioral observations and EEG. Here, we present the results of the cardiovascular study and, in particular, the implemented cardiovascular measurement methods regarding chicken embryos that were designed for investigation of the time point at which chicken embryos are able to respond to a noxious stimulus with a nociceptive cardiovascular response. The corresponding results of the EEG measurements and behavioral observations and the implemented techniques will be presented in further publications.

## 2. Materials and Methods

### 2.1. Animals

Fertilized Lohman Selected Leghorn chicken eggs were obtained from the TUM Animal Research Center (Thalhausen) and stored at 15 °C. Embryonic day (ED) 0 was considered as the day when eggs were transferred to the incubator (Favorit-Olymp 192 Spezial, HEKA-Brutgeräte, Rietberg, Germany). The eggs were incubated for 7 to 18 days at 37.8 °C and 55% humidity and turned six times a day until they were fenestrated.

At ED3 of incubation, the eggshell was fenestrated [24]. For this purpose, the egg was placed horizontally for at least two minutes, and then 5 to 7 mL albumen were withdrawn from the apical pole of the egg using a 5 mL syringe and an 18 G needle. The top of the egg was then covered with tape. A hole was cut in the shell, and the vitality of the embryo was verified. Next, 0.5 mL penicillin-streptomycin (10,000 units penicillin, 10 mg streptomycin/mL, P4333-100 mL Sigma-Aldrich, St. Louis, MI, USA) was added; the egg was then resealed with cling film and was further incubated in a horizontal position. The vitality of the embryos was checked daily until the end of the experiment. Experiments were conducted between 9:00 a.m. and 7:00 p.m. so that the variance in the age of the embryos within an ED was limited to a maximum of 10 h.

### 2.2. Experimental Design

This study was exploratory and was not preceded by an a priori power analysis. At ED12 to ED18, n = 10 embryos of each ED were measured. Due to higher losses in younger embryos, group sizes of n = 6 (ED9) and n = 3 (ED7) embryos were chosen. Furthermore, to study the effect of local anesthesia, n = 6 ED18 embryos were used.

Experiments were performed under standardized conditions in a specially designed heating chamber equipped with a heating lamp (ARTAS GmbH, Arnstadt, Germany) and an air humidifier (HU4811/10 Series 2000, Philips, Amsterdam, The Netherlands). The eggs were placed on a heating mat (ThermoLux, Witte + Sutor GmbH, Murrhardt, Germany) in a bowl filled with warmed Armor Beads (Lab Armor Beads™, Sheldon Manufacturing, Cornelius, NC, USA). The mean temperature and mean humidity during all experiments were 37.7 °C ± 0.8 and 55.5% ± 4.3, respectively.

**Figure 1:**
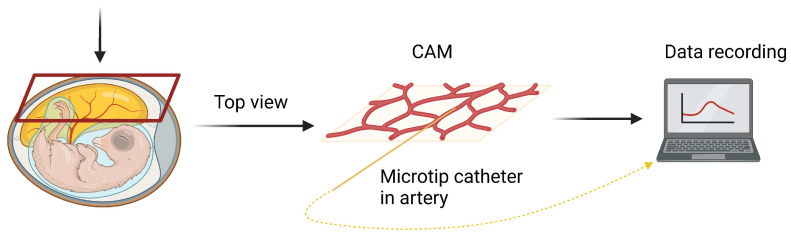
**Schematic illustration of the experimental setup.** A microtip catheter was inserted into a side branch of the chorioallantoic artery, and the values of blood pressure and heart rate (HR) were recorded every four seconds (created with BioRender.com).

A schematic representation of the experimental setup is shown in Figure 1. First, the cling film was removed from the egg, and the shell was carefully opened to the level of the chorioallantoic membrane (CAM). Using a microscope (Stemi SV6, Zeiss, Oberkochen, Germany), the allantoic and amniotic membranes were opened over the head of the embryo, avoiding any large vessels so that the beak could be reached in the further course of the experiment. A side branch of the chorioallantoic artery was prepared, temporarily ligated to avoid blood loss, and incised with microsurgical scissors. A microtip catheter (FISO-LS Fiber Optic Pressure Sensor, FOP-LS-PT9-10, FISO Technologies Inc., Quebec, QC, Canada) was then inserted into the vessel and fixed in place with a ligature. Systolic (SAP), diastolic (DAP) and mean arterial pressure (MAP) as well as HR were recorded continuously every four seconds (PLUGSYS module, EIM-B, EIM-A, HAEMODYN Software v 2.0, Hugo Sachs Elektronik-Harvard Apparatus GmbH, March-Hugstetten, Germany, Evolution Software v 2.1.6.0, FISO Technologies Inc., Quebec, QC, Canada). The beak of the embryo was carefully placed on a Desmarres lid retractor. For younger embryos at ED7 and ED9, the beak was carefully placed on a custom-made wire loop.

After implementation of the catheter, a two-minute waiting period followed. Then, two mechanical stimuli were applied at the base of the beak. In randomized order, a noxious mechanical stimulus was applied with a surgical clamp (*Pinch*), and a light touch (*Touch*) was applied as a negative control. The two stimuli were delivered five minutes apart to allow the parameters to return to the baseline between the stimuli. After the second stimulus, measurements were continued for five more minutes. The measurement time between the two stimuli and after the second stimulus was reduced from five to three minutes for embryos at ED13 and younger due to the increasing sensitivity of the organism.

For the *Pinch*, a surgical clamp was applied to the base of the beak and squeezed. For *Touch,* the beak was only lightly touched with the surgical clamp. For both stimuli, a mosquito clamp was used for ED12 to ED18 embryos. For embryos at ED7 and ED9, the surgical clamp was too large, and microsurgical forceps were used instead for both stimuli. To ensure comparability, the stimuli were always applied by the same person. In the further course of the study, an analgesia meter (BIO-RP-M, BioSeb, Vitrolles, France) with customized tips of the mosquito clamp was used to monitor the pressure applied by the mechanical stimuli.

To verify whether the measured cardiovascular responses could be classified as nociceptive responses, a local anesthetic was applied to n = 6 ED18 embryos before stimulation. For this purpose, after the preparation and placement of the microtip catheter, 0.02 mL of lidocaine 2% (Xylocitin^®^ 2%, Mibe GmbH Arzneimittel, Brehna, Germany) were injected into the upper and lower beak using a 30 G needle (*ED18 w/Lido Touch* and *Pinch*). The measurements were carried out following the same experimental protocol as for other ED14 to ED18 embryos with the exception that a waiting period of three minutes was added prior to the measurement. During this time, blood pressure and HR were monitored for the occurrence of side effects of lidocaine, such as bradycardia, arrhythmia or hypotension. As a comparison group without lidocaine, the already measured ED18 embryos were used (*ED18 w/o Lido Touch* and *Pinch*).

Immediately after the end of the experiments, the embryos were euthanized by intravenous injection of pentobarbital sodium (Narcoren^®^, 16 g/100 mL, Boehringer Ingelheim Vetmedica GmbH, Ingelheim am Rhein, Germany; ED7–ED12: 0.1 mL; ED13–ED19: 0.2 mL) followed by decapitation.

### 2.3. Analysis

SAP, DAP, MAP and HR were recorded every four seconds. For the evaluation of the reactions to the stimuli, the means of MAP and HR were calculated over one minute before (=baseline) and one minute after the respective stimulus. To avoid any influence of the approach of the clamp, the 15 s immediately before the respective stimuli were introduced were not included as part of the baseline. In embryos showing a hyperacute decrease in HR with a subsequent increase in HR after *Pinch*, the decrease was not included in the calculation and was evaluated separately to avoid negation of opposite reactions. The deviation of the response after the stimulus (*Pinch*/*Touch*) as a percentage of the baseline value was then calculated. Differences in the percent changes to the baseline in MAP and HR after *Pinch* and *Touch* were tested for statistical significance. For normally distributed data, a paired *t*-test (two-tailed) was used. For data that failed the normality test, a Wilcoxon signed-rank test (two-tailed) was performed. For the comparison of multiple groups, either a one-way ANOVA (normally distributed) or a Kruskal—Wallis test (not normally distributed) was used. Additional information on statistical metrics can be found in Table S1.

## 3. Results

### 3.1. Increasing Arterial Pressure and Evolution of HR during Embryonic Development of the Chicken

SAP, DAP and MAP in the chorioallantoic artery and HR were recorded over one minute at ED7, ED9 and EDs 12 to 18. SAP, DAP and MAP increased with the age of the embryos (Table 1). ED7 showed the lowest MAP with a value of 2.08 mmHg ± 0.40, and ED18 showed the highest MAP with a value of 17.28 mmHg ± 3.04.

### 3.2. Increase in MAP in Response to a Noxious Stimulus

**Figure 2:**
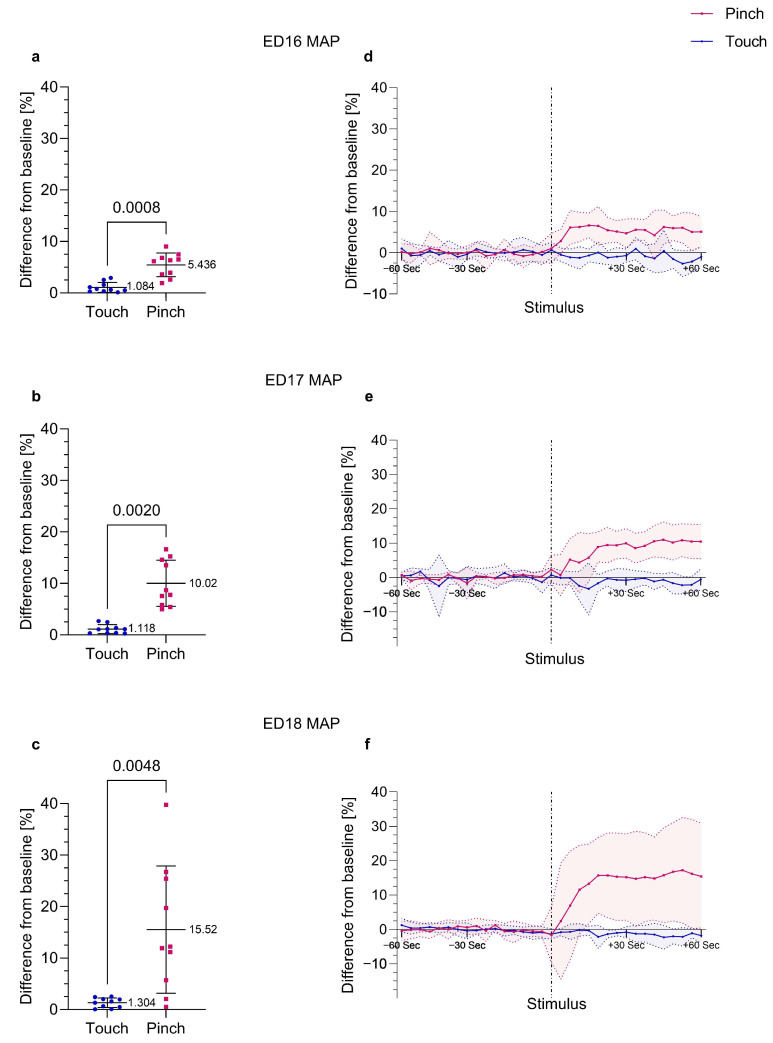
**Percent change in MAP post *Touch* and *Pinch.*** Embryos at EDs 16 to 18 (n = 10) received a noxious mechanical stimulus (*Pinch*) and a light touch as a negative control (*Touch*) at the base of the beak in randomized order. (**a**–**c**) Percent change from baseline MAP after *Pinch* compared to *Touch*. Displayed as the mean ± standard deviation. Paired *t*-test (normally distributed: (**a**,**c**)) or Wilcoxon signed-rank test (not normally distributed: (**b**)). Mean and *p* values shown; (**a**): *p* = 0.0008, (**b**): *p* = 0.0020, (**c**): *p* = 0.0048. (**d**–**f**) Percent change from the baseline mean value of MAP over time; values recorded every four seconds for one minute before and one minute after stimulation (*Pinch* and *Touch*); values shown as the mean ± standard deviation (shaded).

The response of MAP to a noxious mechanical stimulus at the base of the beak (*Pinch*) was compared to the response to a light touch at the base of the beak as a negative control (*Touch*) in embryos between EDs 7 and 18. As shown in Figure 2, a significant increase in MAP was observed as a reaction to *Pinch* in embryos on ED16 (*p* = 0.0008, *r* = 0.857), ED17 (*p* = 0.0020, *r* = 0.627) and ED18 (*p* = 0.0048, *r* = 0.778). ED18 embryos showed the strongest response in MAP, with an increase of 15.52% ± 12.36 from the baseline. In comparison, a deviation from the baseline of only 1.30% ± 0.94 was detected in response to *Touch* on ED18. In embryos at ED7, ED9 and EDs from 12 to 15, no significant differences between the MAP responses to *Pinch* and *Touch* were detected, which can be seen in Figures S1 and S2.

### 3.3. Changes in HR in Response to a Noxious Stimulus

**Figure 3:**
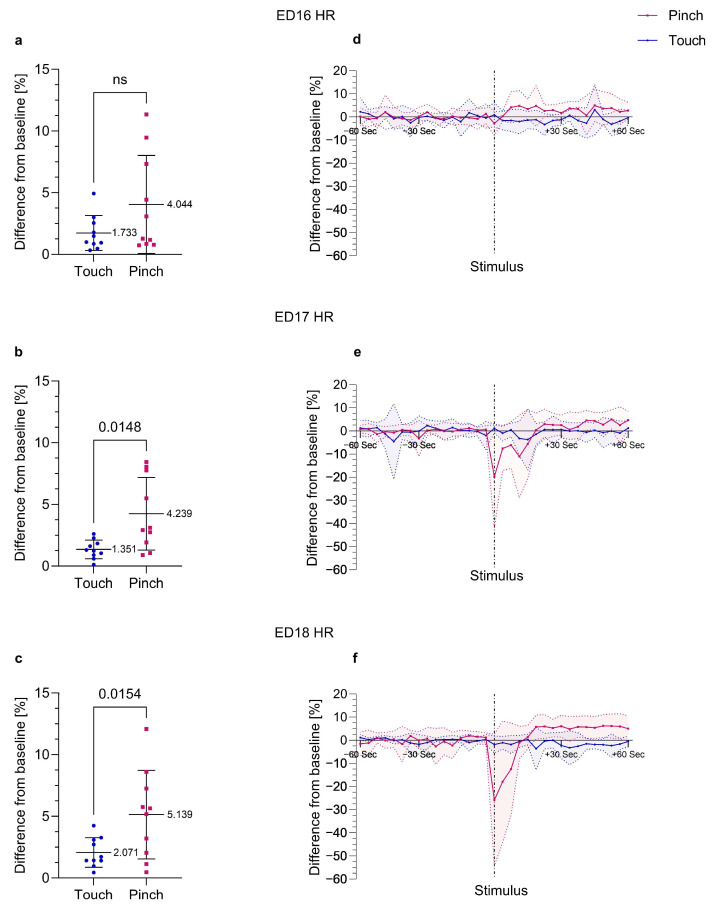
**Percent change in HR post *Touch* and *Pinch*.** Embryos at EDs 16 to 18 (n = 10) received a noxious mechanical stimulus (*Pinch*) and a light touch as a negative control (*Touch*) at the base of the beak in randomized order. (**a**–**c**) Percent change from baseline HR after *Pinch* compared to *Touch*. Displayed as the mean ± standard deviation. Paired *t*-test (normally distributed: (**b**,**c**)) or Wilcoxon signed-rank test (not normally distributed: (**a**)). Mean and *p* values shown; (**b**): *p* = 0.0148, (**c**): *p* = 0.0154; ns = no significant difference between the groups (**a**). (**d**–**f**) Percent change from the baseline mean value in HR over time; values recorded every four seconds for one minute before and one minute after stimulation (*Pinch* and *Touch*); values shown as the mean ± standard deviation (shaded).

Regarding HR, two reaction patterns were observed, particularly in ED17 and ED18 embryos. In some embryos, HR immediately increased after *Pinch*. In other embryos, a hyperacute decrease in HR followed by an increase was observed in response to *Pinch*, as shown in Figure 3d–f. A change in HR of at least −15% with a subsequent increase of at least 5% from the baseline mean value after *Pinch* was observed in 80% of ED18 embryos and in 30% of ED17 embryos and was not detected after *Touch*. In embryos at ED18, HR decreased by up to −48.54% ± 19.71 over 9.50 s ± 6.02 on average after *Pinch*. At ED17, these embryos showed a decrease in HR by up to −41.87% ± 8.32 over 16.00 s ± 6.93 on average after *Pinch*. Simultaneous with the hyperacute decrease in HR, a slight decrease in MAP was also observed, particularly when the decrease in HR was large. In embryos at ED15 and ED16, the observations were inconsistent and could not be clearly distinguished from physiological variations in HR. In younger embryos, no hyperacute decrease in HR was observed in response to *Pinch*.

Significant increases in HR in response to *Pinch* compared to *Touch* were detected in embryos at ED17 (*p* = 0.0148, *r* = 0.708) and ED18 (*p* = 0.0154, *r* = 0.705) (Figure 3a–c). Embryos at ED18 showed the largest increase in HR after *Pinch,* with a deviation of 5.14% ± 3.60 from the baseline, compared to a deviation of only 2.07% ± 1.20 from the baseline after *Touch*. At ED7, ED9 and EDs 12 to 16, no significant changes in HR were observed, as shown in Figures S3 and S4.

### 3.4. Reduction of Cardiovascular Response by Local Anesthesia

**Figure 4:**
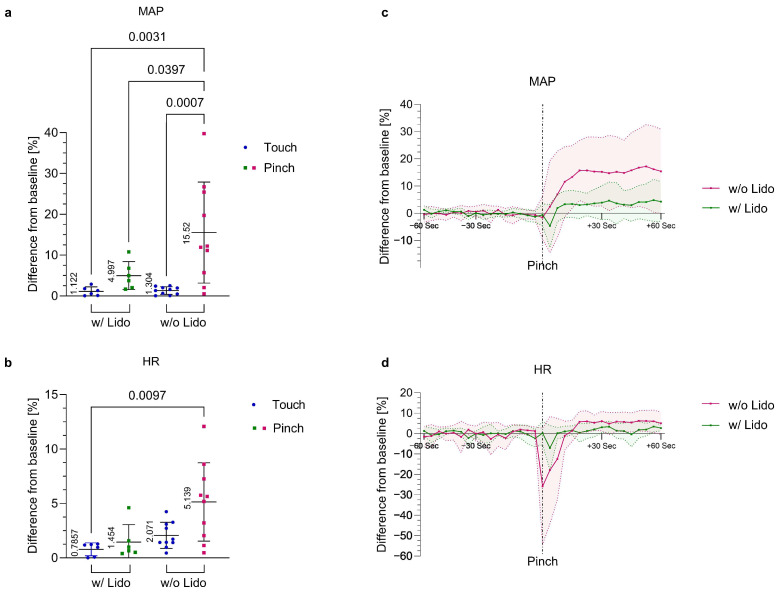
**Local anesthesia control group. Percent change in MAP and HR post *Touch* and *Pinch*.** ED18 embryos either received a lidocaine injection (*ED18 w/Lido*; n = 6) or no lidocaine injection (*ED18 w/o Lido*; n = 10) at the base of the beak prior to stimulation (*Touch* and *Pinch*). (**a**) Percent change from baseline MAP after *Pinch* in the group without lidocaine (*ED18 w/o Lido Pinch*) compared to *ED18 w/o Lido Touch*, *ED18 w/Lido Touch* and *ED18 w/Lido Pinch*. Displayed as the mean ± standard deviation. One-way ANOVA (normally distributed); mean and *p* values shown. (**b**) Percent change from baseline HR after *Pinch* in the group without lidocaine (*ED18 w/o Lido Pinch*) compared to *ED18 w/o Lido Touch*, *ED18 w/Lido Touch* and *ED18 w/Lido Pinch*. Displayed as the mean ± standard deviation. Kruskal—Wallis test (not normally distributed); mean and *p* values shown. (**c***,***d**) Percent change from the baseline mean value in MAP and HR after *Pinch* over time; values recorded every four seconds for one minute before and one minute after stimulation (*ED18 w/o* or *w/Lido Pinch*); values shown as the mean ± standard deviation (shaded).

The application of the local anesthetic lidocaine (*Lido*) at the base of the beak prior to stimulation significantly reduced the MAP increase in response to *Pinch* in embryos at ED18. Compared to the group without local anesthesia (*ED18 w/o Lido*), which showed an increase of 15.52% ± 12.36 post *Pinch*, the increase in MAP was reduced to 5.00% ± 3.42 in the group that received lidocaine (*ED18 w/Lido*). As represented in Figure 4a,c, the *ED18 w/o Lido Pinch* group showed the largest increase in MAP in response to *Pinch*, exceeding those of the *ED18 w/o Lido Touch* (*p* = 0.0007), *ED18 w/Lido Touch* (*p* = 0.0031) and *ED18 w/Lido Pinch* (*p* = 0.0397) groups, with an effect size of *η*^2^ = 0.467.

The changes in HR in response to *Pinch* were slightly reduced by the application of lidocaine. However, a significant difference in HR was observed only between *ED18 w/o Lido Pinch* and *ED18 w/Lido Touch* (*p* = 0.0097), as displayed in Figure 4b. In the group treated with lidocaine (*ED18 w/Lido*), no embryo showed a hyperacute change in HR of −15% with a subsequent increase of 5% from the baseline mean value after the stimulus, but this reaction pattern was observed in 80% of the embryos in the *ED18 w/o Lido Pinch* group. A slight decrease in HR after *Pinch* was also observed in the local anesthetic group (*ED18 w/Lido Pinch*), but this decrease could not be distinguished from physiological variations in HR (Figure 4d).

## 4. Discussion

This study successfully developed methods to record blood pressure and HR in chicken embryos between EDs 7 and 18. Cardiovascular changes in response to a noxious mechanical stimulus at the base of the beak were investigated with the aim of identifying the onset of nociception during embryonic development in chickens.

Although there are many well-established noninvasive methods for determining HR in chicken embryos [25,26,27], direct intra-arterial measurement is the gold standard for recording blood pressure [28]. In the past, blood pressure in chicken embryos was measured using glass capillaries or needle catheters inserted into an embryonic artery [29,30,31]. Corresponding to prior descriptions in the literature [29,30,31], an increase in arterial blood pressure with increasing age of the embryos was observed in the present study, but there were no major differences in HR between the EDs. Thus, the optical measurement of arterial blood pressure and HR with a microtip catheter represents a reliable method for invasive measurement of blood pressure and HR in chicken embryos. However, insertion of the catheter was particularly challenging at ED7 and ED18 due to the small size of the chorioallantoic vessels at ED7 and the beginning regression of the chorioallantoic vessels at ED18.

Because self-reporting is not possible in animals, it is difficult to evaluate their pain perception [22]. On the other hand, nociceptive reactions to a noxious stimulus can be measured [13]. The recording of cardiovascular parameters is well suited to the clinical evaluation of nociception in animals, including birds [32,33]. In the present study, the acquisition of cardiovascular parameters could be established for chicken embryos between EDs 7 and 18. Blood pressure and HR are mainly influenced by the autonomic nervous system [34]. Transmission of a noxious stimulus to the central nervous system results in activation of the sympathetic nervous system, which usually leads to an increase in blood pressure and HR [34]. Therefore, recording cardiovascular variables is considered the gold standard for the detection of nociception under anesthesia [35].

As a means of assessing the cardiovascular response of the chicken embryo to a noxious mechanical stimulus at the base of the beak, MAP was found to be the most sensitive parameter in the present study. Significant differences in MAP between *Pinch* and *Touch* were detected earliest on ED16 (Figure 2), whereas significant changes in HR were only observed in ED17 and ED18 embryos (Figure 3). Effect sizes were high, indicating the clinical relevance of the findings. Although there was a distinct increase in MAP in response to *Pinch* that reached over 10% deviation from the baseline in ED17 and ED18 embryos, the changes in HR were variable, and there were not necessarily any associations between changes in MAP and HR. Similar observations have been reported in adult chickens [36]. MAP has also been described in other studies concerning nociceptive responses in mammals as the most sensitive indicator of nociception [34,37].

A prerequisite for cardiovascular response to external stimuli is functional regulation of the cardiovascular system. Blood pressure in the chicken embryo is mainly regulated by the sympathetic nervous system [38]. The adrenergic tone in the cardiovascular system is considered to be present from a point in time that is halfway through the incubation period [39,40]. Therefore, the sympathetic influence on blood pressure is expected to be functional from approximately ED10 [39]. In the heart, adrenergic and cholinergic receptors are already functional on ED4 [41]. Changes in HR due to alterations in environmental conditions such as oxygen levels and temperature have already been observed on ED3 [42]. In the present study, significant changes in HR after a noxious stimulus was introduced were not observed until ED17 (Figure 3).

Another prerequisite for the assessment of a nociceptive response is functioning stimulus transmission. Despite some differences in the nervous system, the processing of noxious stimuli in birds is comparable to that in mammals [13]. C-fibers and A-delta fibers have been found in chickens, innervating the beak, nasal and buccal mucosa as well as the legs [11,43]. High-threshold mechanothermal nociceptors are polymodal and respond to mechanical lesions, elevated temperatures and chemical insult [13]. It is believed that injuries to the beak can be highly painful for the bird [43], because the beak tip is an intensely innervated area [44], and both the upper and the lower portions of the beak contain nociceptors [45]. Reflective reactions such as movements of the head to mechanical and thermal stimuli and to needle punctures appear for the first time in the skin area of the beak on ED7 [46]. Therefore, in the present study, the application of a noxious stimulus to the base of the beak was chosen to evoke the highest possibility for a nociceptive response.

Regarding HR, irregularities appeared spontaneously over the whole measurement period, even at the baseline. Mainly short decelerations in HR were observed, whereas MAP was not affected. It has already been reported in several publications that HR irregularities physiologically occur at the end of the second week of incubation [47,48,49,50]. Nevertheless, the HR irregularities did not have a great influence on the calculation of the mean. Minor changes in DAP corresponded to the HR irregularities, but the analysis showed that MAP was not affected. In contrast to physiological variations in HR, a hyperacute decrease in HR with a subsequent increase could be clearly identified as a response to *Pinch* in 30% of ED17 and 80% of ED18 embryos. This reaction pattern could be distinguished from physiological variations in HR by the finding that after *Pinch*, HR decreased by at least −15%, followed by a sustained increase in HR by at least 5% from the baseline mean value. The decrease in HR after *Pinch* was also accompanied by a short decrease in MAP followed by an increase. A decrease in HR as a reaction to a noxious stimulus has been reported in adult chickens [36] and in mammals [51,52] and may be due to a vasovagal reflex to a noxious stimulus [53]. However, only a few individual embryos showed a hyperacute decrease in HR after *Pinch*, which shows that the response in HR to a noxious stimulus varies among individuals. Variable responses in HR after a noxious stimulus have also been described in adult chickens [36]. Considering these different observations regarding HR, it is difficult to draw conclusions about the presence of nociception. Thus, HR should not be used as a single parameter for evaluating a nociceptive response in chicken embryos; however, MAP was shown to be a more sensitive parameter in the present study.

In addition to a nociceptive response, it must also be considered that the measured cardiovascular changes may be induced by other factors that influence the autonomic nervous system [54] or by embryonic movements. Especially in birds, physiological variables can be influenced by many external factors, such as temperature, light, or handling [54]. A correlation between fetal movements and HR irregularities has been described in human fetuses [55]. In the present study, movements of the embryo induced minor variations in HR and DAP, but MAP was not affected. No sustained increase in MAP and HR as observed in response to *Pinch* could be attributed to movements.

Infiltration anesthesia at the base of the beak could be used to verify that the measured changes in MAP and HR may be classified as a nociceptive response and were not caused by embryonic movements or factors that influence the autonomic nervous system. The application of local anesthetics is one of the best methods to prevent the generation and transmission of nociceptive impulses [56]. These anesthetics act by blocking sodium channels in the nerve axon [54]. The application of lidocaine or bupivacaine has been described as an effective method of analgesia in birds [57]. However, the time of onset of action and the duration of action are not defined for birds [54]. In the present study, lidocaine was used because it has a rapid onset of action in mammals [56], as well as a short onset of action for spinal anesthesia in chickens [58]. Given that higher sensitivity to local anesthetics is expected in birds than in mammals [59], embryos were intensively monitored for the occurrence of toxic effects. No signs of side effects such as bradycardia, arrhythmia or hypotension were observed in the tested embryos. Because the increase in MAP was significantly reduced by the injection of lidocaine (Figure 4), the cardiovascular reactions to *Pinch* in the embryos that did not receive local anesthesia might be interpreted as a nociceptive response to the noxious stimulus. A limitation and possible explanation for the incompletely suppressed reaction in some embryos was that injection into the beak of the moving embryo was challenging, and infiltration of the entire beak area could not always be assured. It must be mentioned that the present study was exploratory and the size of the group receiving local anesthesia was rather small. Further investigations would need to be performed to verify the effect of local anesthesia and to ultimately exclude other factors as the cause of the measured cardiovascular changes. However, assuming that it is a nociceptive response, further studies regarding anesthesia and analgesia protocols are necessary to provide improved animal welfare for chicken embryos in research. Cardiovascular variations are commonly used to determine the need for analgesia or sedatives [23], and thus far, there are no EU-wide regulations regarding anesthesia and analgesia for chicken embryos in research.

Although no significant difference between *Pinch* and *Touch* was reached at ED15 in MAP (Figure S1f) and HR (Figure S3f), individual responses could be observed. Occasionally, embryos at ED15 showed reactions in MAP (Figure S2f) and HR (Figure S4f) after *Pinch*. The measurements of these embryos were performed late in the day. The development of the embryos could therefore have been more advanced compared to embryos examined in the morning. In addition, embryonic development can be influenced by various factors, and some embryos might progress faster in development than others [39]. Therefore, it must be assumed that a nociceptive cardiovascular response is possible in individual embryos at ED15.

A limitation of the study was that intra-arterial measurement of blood pressure and HR is an invasive method. The measurements had to be performed on the fenestrated egg, making it necessary to open the egg membranes. Because chicken embryos are highly sensitive to external factors [29,42], special care was taken to maintain standardized environmental conditions and to avoid blood loss during preparation. In some embryos, severe bradycardia and hypotension were observed, or HR frequently decreased to zero. These embryos had to be excluded from the analysis because reliable measurements could not be completed. At ED7, reaching the beak was challenging, and a measurement could only be performed in three embryos; severe arrhythmias affecting MAP were observed. The microtip catheter is designed to measure low pressures, but the measurement accuracy of 2 mmHg, according to the manufacturer, reached its limits with the occurrence of extremely low blood pressure in ED7. The results from ED7 should therefore be interpreted with caution.

## 5. Conclusions

In conclusion, significant differences and large effect sizes in a cardiovascular response to a mechanical noxious stimulus at the base of the beak compared with a light touch at the base of the beak were detected in chicken embryos on EDs 16 to 18. For individual embryos, cardiovascular changes after the introduction of a noxious mechanical stimulus have already been observed on ED15. MAP was found to be the most sensitive parameter in the present study, whereas variable observations were made regarding HR. Infiltration anesthesia with the local anesthetic lidocaine (2%) significantly reduced the reactions of MAP to a noxious mechanical stimulus at the base of the beak in ED18 embryos, indicating that the measured cardiovascular changes may be interpreted as nociceptive responses. However, it must be mentioned that this was an exploratory study with a correspondingly small group size. To assess response to a noxious stimulus, a multiparametric approach should be adopted and several parameters should be assessed in their entirety [60]. Thus, to properly evaluate a nociceptive response in the chicken embryo, other parameters, such as movement analysis, should be taken into account in addition to hemodynamic parameters.

## Figures and Tables

**Table 1:** Systolic arterial pressure (SAP), diastolic arterial pressure (DAP), mean arterial pressure (MAP) and HR at embryonic day (ED) 7 (n = 3), ED9 (n = 6), and ED12 to ED18 (n = 10). Values are shown as the mean ± standard deviation.

	ED7	ED9	ED12	ED13	ED14	ED15	ED16	ED17	ED18
SAP (mmHg)	3.50 ± 0.65	6.04 ± 1.46	9.19 ± 1.32	9.88 ± 1.52	13.02 ± 1.60	16.54 ± 3.04	21.44 ± 2.78	24.46 ± 5.50	24.65 ± 4.36
DAP (mmHg)	1.07 ± 0.36	1.98 ± 1.10	2.20 ± 1.12	2.96 ± 0.61	3.95 ± 1.14	5.69 ± 1.82	7.80 ± 2.16	10.77 ± 3.53	11.43 ± 2.43
MAP (mmHg)	2.08 ± 0.40	3.44 ± 1.24	4.83 ± 1.05	5.52 ± 0.79	7.32 ± 1.26	10.11 ± 2.45	13.73 ± 2.38	16.79 ± 4.21	17.28 ± 3.04
HR (bpm)	128.97 ± 15.40	147.57 ± 9.03	159.08 ± 26.64	146.61 ± 19.99	179.10 ± 35.06	154.33 ± 33.12	151.35 ± 36.44	179.08 ± 29.18	176.07 ± 35.75

## Data Availability

Raw data are available from the corresponding author upon reasonable request.

## Supplementary Materials

The following supporting information can be downloaded at: https://www.mdpi.com/article/10.3390/ani13172710/s1, Figure S1: Percent change in MAP post *Touch* and *Pinch*; Figure S2: Percent change from the baseline mean value in MAP over time; Figure S3: Percent change in HR post *Touch* and *Pinch*; Figure S4: Percent change from the baseline mean value in HR over time; Table S1: Statistical metrics.
