# Supplementary material for: Nociception in Chicken Embryos, Part I: Analysis of Cardiovascular Responses to a Mechanical Noxious Stimulus
Source: Animals (Basel). 2023 Aug 25;13(17):2710. doi: 10.3390/ani13172710 (PMC10486618; doi:10.3390/ani13172710)
Supplement: Supplementary file 1 [file animals-13-02710-s001.zip › Supplementary information_Part_I_final.pdf]

Supplementary information

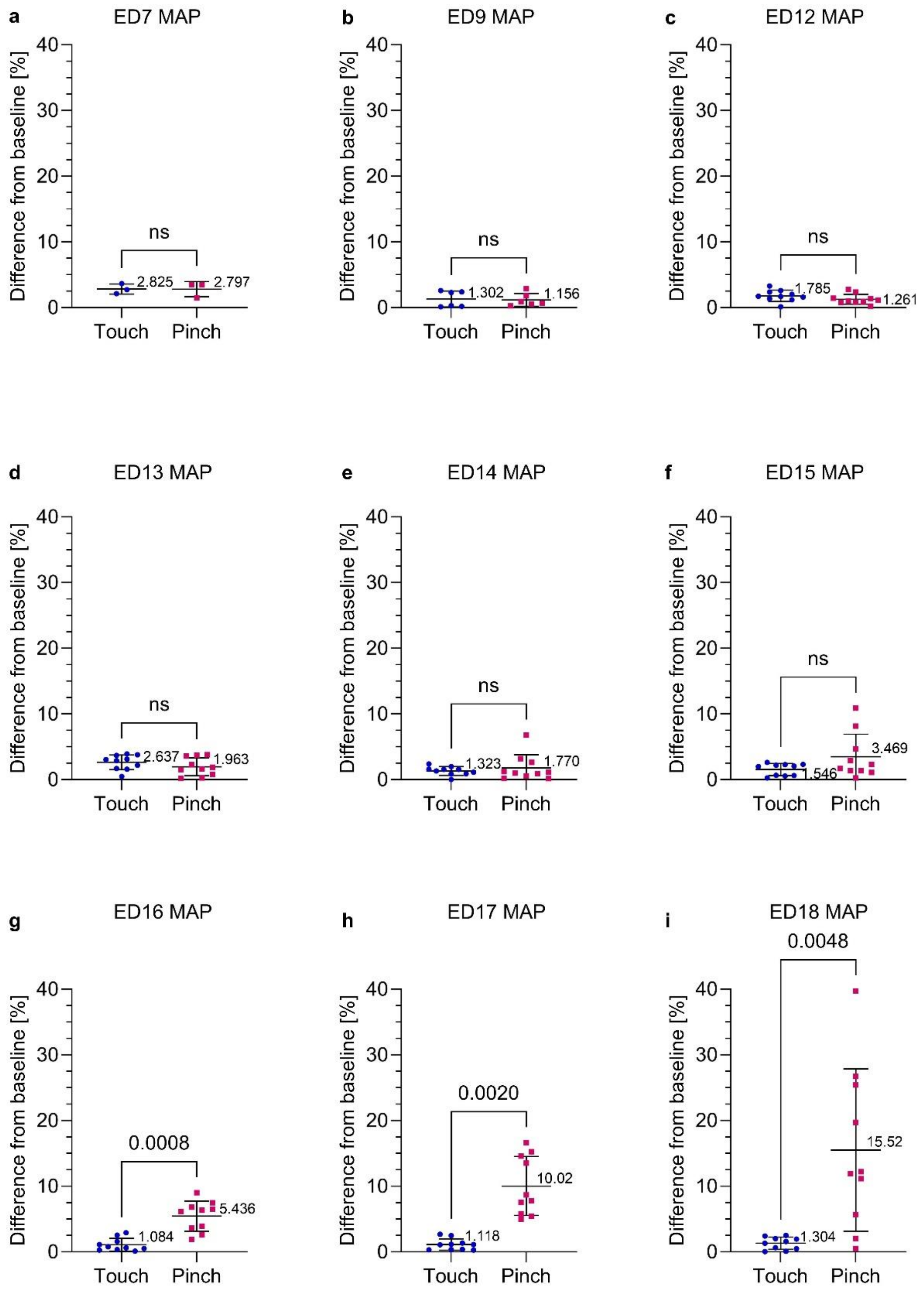

Figure S1. Percent change in MAP post *Touch* and *Pinch*. a-i Embryos at embryonic day (ED) 7 (n=3), ED9 (n=6) and EDs 12 to 18 (n=10) received a mechanical noxious stimulus (*Pinch*) and a light touch as control (*Touch*) at the base of the beak in randomized order. Displayed as the mean ± standard deviation. Paired t-test (normally distributed: c, d, g and i) or Wilcoxon signed-rank test (not normally distributed: a, b, e, f and h). Mean and p values shown; g: p=0.0008, h: p=0.0020 and i: p=0.0048; ns = no significant difference between the groups.

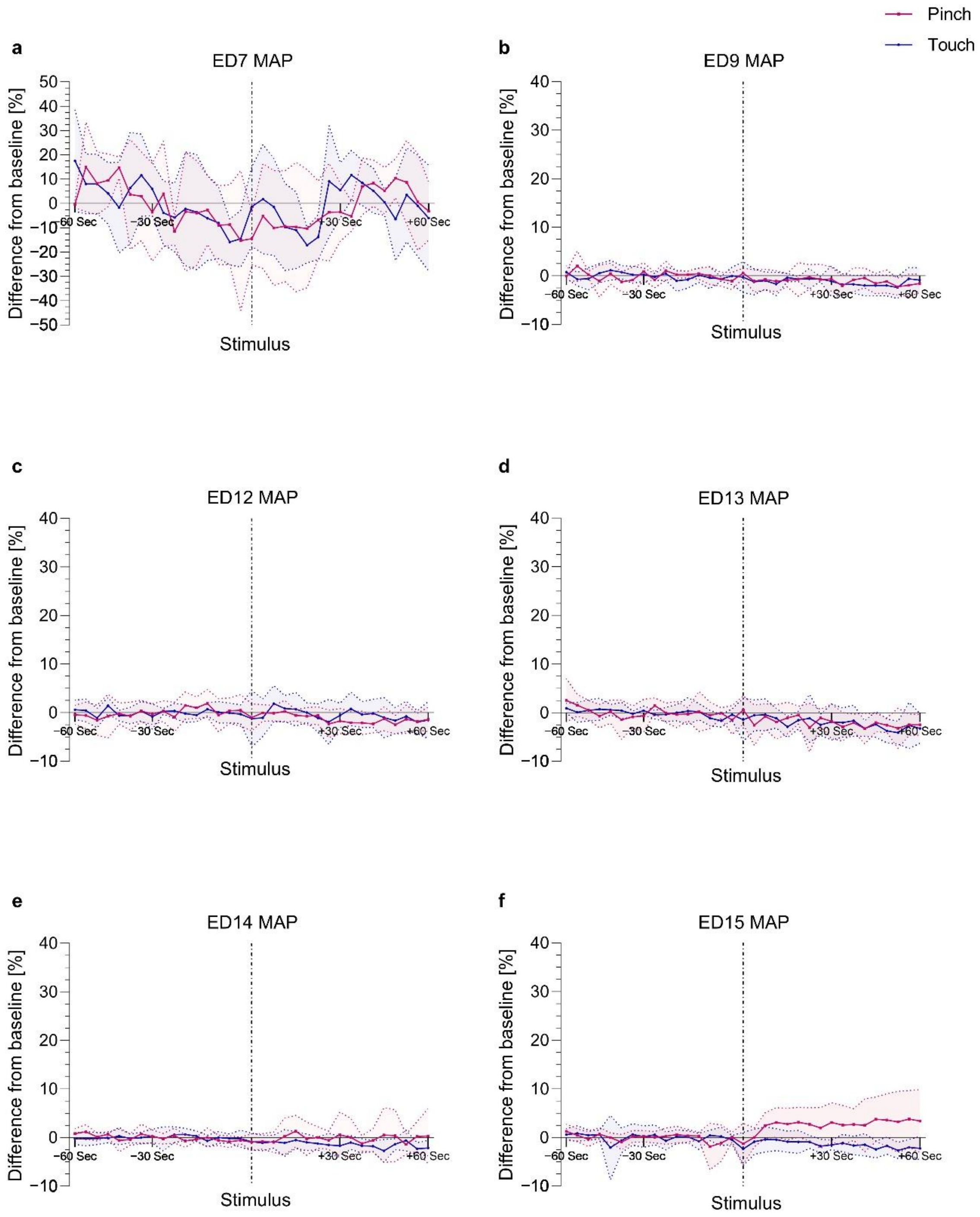

Figure S2. Percent change from the baseline mean value in MAP over time. a-f Embryos at ED7 (n=3), ED9 (n=6) and EDs 12 to 15 (n=10) received a mechanical noxious stimulus (*Pinch*) and a light touch as control (*Touch*) at the base of the beak in randomized order. Values were recorded every four seconds for one minute before and after stimulation (*Touch* and *Pinch*). Values are shown as the mean ± standard deviation (shaded).

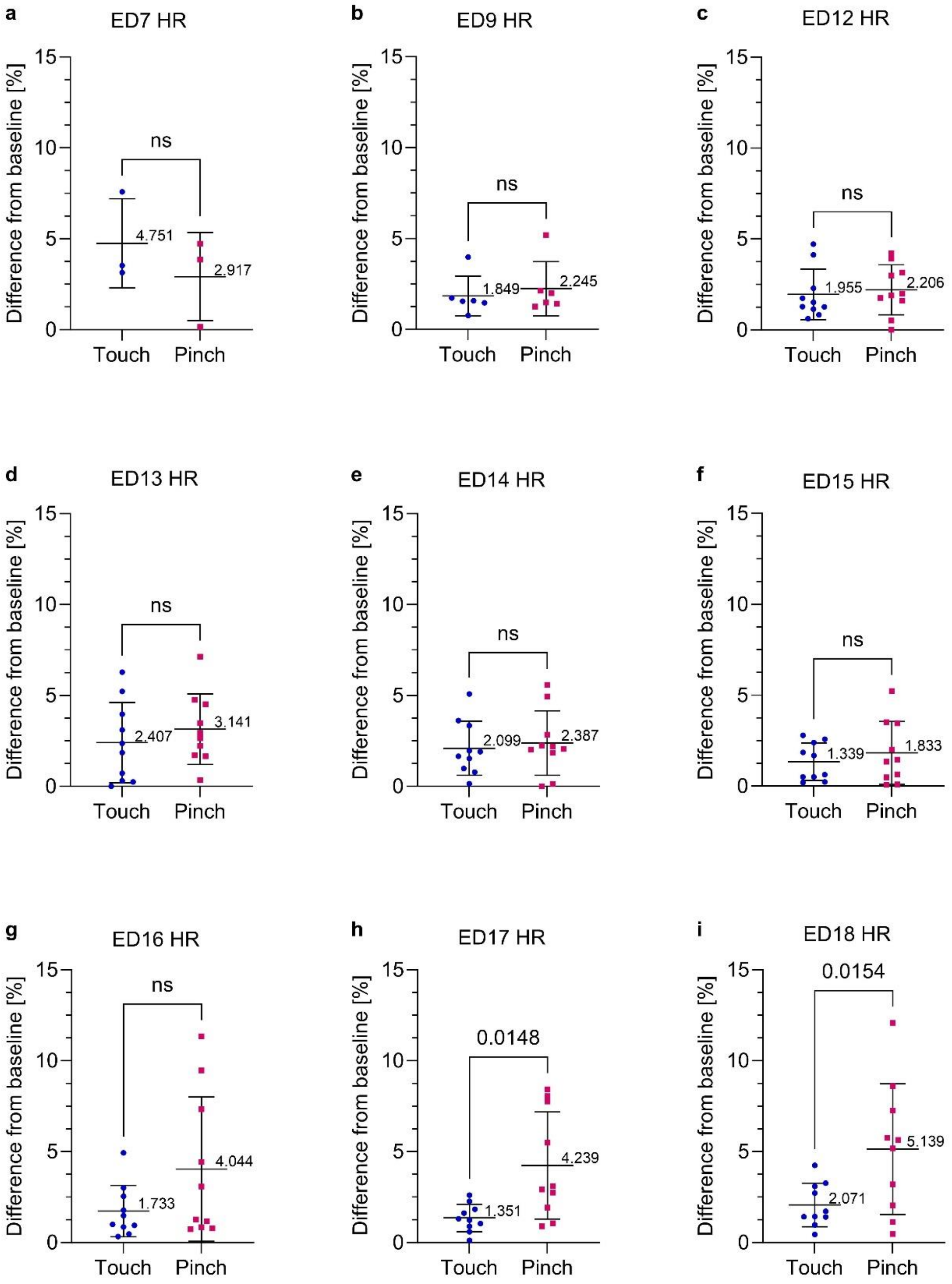

Figure S3. Percent change in HR post *Touch* and *Pinch*. a-i Embryos at ED7 (n=3), ED9 (n=6) and EDs 12 to 18 (n=10) received a mechanical noxious stimulus (*Pinch*) and a light touch as control (*Touch*) at the base of the beak in randomized order. Displayed as the mean ± standard deviation. Paired t-test (normally distributed: a, d-f, h and i) or Wilcoxon signed-rank test (not normally distributed: b, c and g). Mean and p values shown; h: p=0.0148 and i: p=0.0154; ns = no significant difference between the groups.

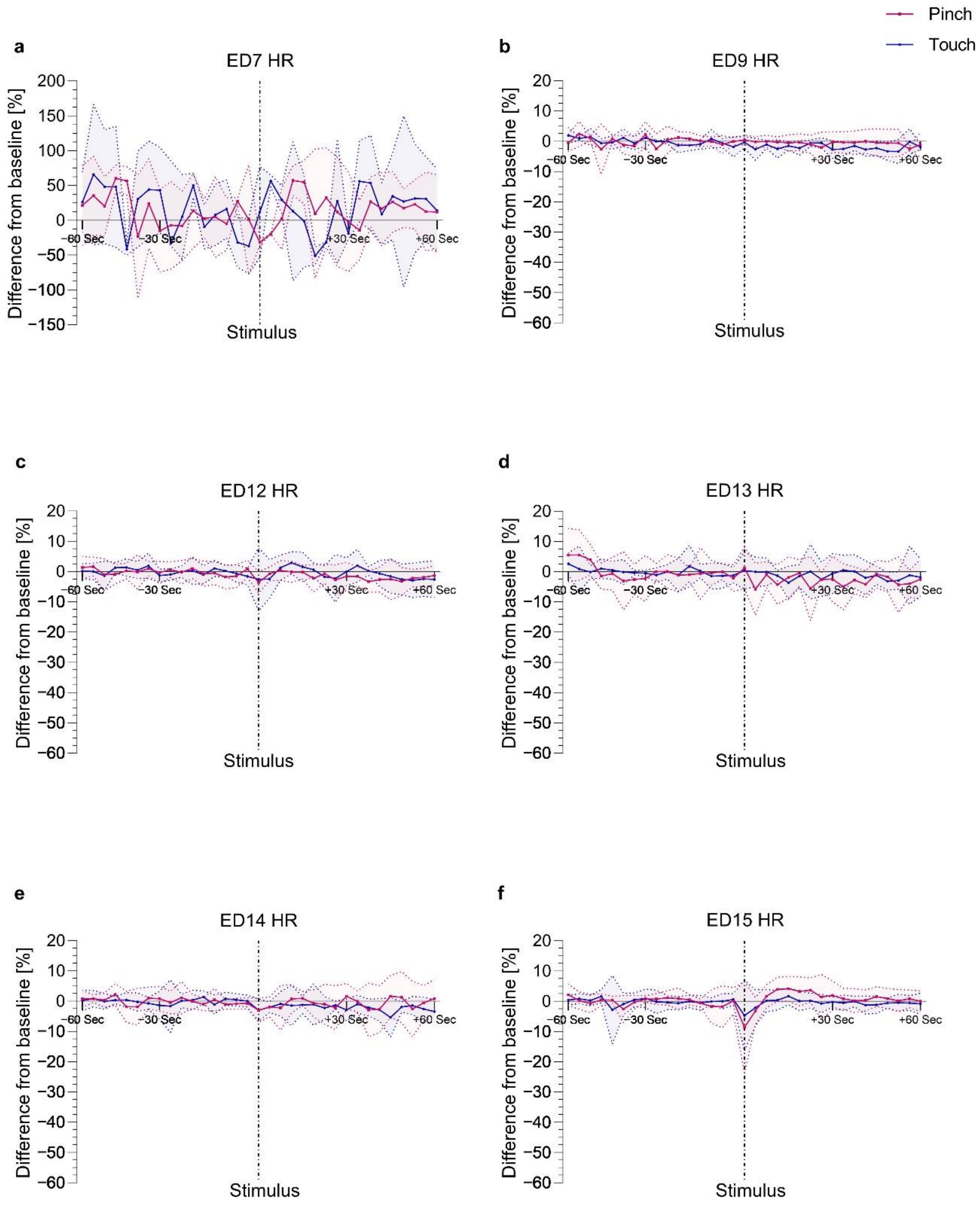

Figure S4. Percent change from the baseline mean value in HR over time. a-f Embryos at ED7 (n=3), ED9 (n=6) and EDs 12 to 15 (n=10) received a mechanical noxious stimulus (*Pinch*) and a light touch as control (*Touch*) at the base of the beak in randomized order. Values were recorded every four seconds for one minute before and after stimulation (*Touch* and *Pinch*). Values are shown as the mean ± standard deviation (shaded).
